# Sorafenib alone vs. sorafenib plus GEMOX as 1^st^-line treatment for advanced HCC: the phase II randomised PRODIGE 10 trial

**DOI:** 10.1038/s41416-019-0443-4

**Published:** 2019-04-04

**Authors:** Eric Assenat, Georges-Philippe Pageaux, Simon Thézenas, Jean-Marie Peron, Yves Bécouarn, Jean-François Seitz, Philippe Merle, Jean-Frédéric Blanc, Olivier Bouché, Mohamed Ramdani, Sylvain Poujol, Hélène de Forges, Marc Ychou, Valérie Boige

**Affiliations:** 1 Institut du Cancer de Montpellier (ICM), Université de Montpellier, Montpellier, France; 2 Centre Hospitalier Universitaire de Montpellier, Université de Montpellier, Montpellier, France; 3 Centre Hospitalier Universitaire de Toulouse, Toulouse, France; 4 Bergonié Institute, Bordeaux, France; 5 Centre Hospitalier Universitaire de Marseille, Marseille, France; 6 Centre Hospitalier Universitaire de Lyon, Lyon, France; 7 Saint-André Hospital, Bordeaux, France; 8 Centre Hospitalier Universitaire de Reims, Reims, France; 9 Centre Hospitalier Universitaire de Béziers, Béziers, France; 10 Gustave Roussy, Villejuif, France

**Keywords:** Chemotherapy, Hepatocellular carcinoma, Randomized controlled trials

## Abstract

**Background:**

Sorafenib remains one major first-line therapeutic options for advanced hepatocellular carcinoma (aHCC), with modest efficacy. We investigated the addition of gemcitabine and oxaliplatin (GEMOX) to sorafenib in aHCC patients.

**Methods:**

Our multicentre phase II trial randomised aHCC first-line patients to sorafenib (400 mg BID) or sorafenib-GEMOX every 2 weeks (1000 mg/m^2^ gemcitabine; 100 mg/m^2^ oxaliplatin). Primary endpoint was the 4-month progression-free survival (PFS) rate.

**Results:**

Ninety-four patients were randomised (sorafenib-GEMOX: *n* = 48; sorafenib: *n* = 46). Median age was 64 years, PS 0 (69%) or 1 (31%), 63% patients had cirrhosis, 29% portal vein thrombosis and 70% extra-hepatic disease. Median duration of sorafenib treatment was 4 months (1–51); median number of GEMOX cycles was 7 (1–16). The 4-month PFS rates were 64% and 61% in the sorafenib-GEMOX and sorafenib arms, respectively; median PFS and OS were 6.2 (95% CI: 3.8–6.8) and 13.5 (7.5–16.2) months, and 4.6 (3.9–6.2) months and 14.8 (12.2–22.2), respectively. The ORR/DCR were 9%/70% and 15%/77% in the sorafenib-GEMOX and sorafenib alone arms, respectively. Main toxicities were (sorafenib-GEMOX/sorafenib) neutropenia (23%/0), thrombocytopenia (33%/0), diarrhoea (18%/9), peripheral neuropathy (5%/0) and hand–foot syndrome (5%/18).

**Conclusions:**

Addition of GEMOX had an inpact on ORR and was well-tolerated as frontline systemic therapy. The benefit on PFS seems moderate; no subsequent study was planned.

## Background

Hepatocellular carcinoma (HCC) is a vascular tumour with poor prognosis. Worldwide, HCC is the fifth most common cancer in men and the seventh in women, and is responsible for more than 600,000 cancer deaths.^1^ This high incidence can mainly be attributed to a high prevalence of hepatitis B virus infection.^2^ Most patients with HCC are not eligible for any potentially curative therapy due to the high burden of the liver disease, extra-hepatic spread, or poor background liver function related to cirrhosis. They are then considered for palliative therapies. Furthermore, HCC is often considered resistant to common cytotoxic chemotherapies. Although doxorubicin was initially considered the agent of choice in advanced HCC, two controlled trials suggested it is associated with poor survival and a modest overall response rate compared with the best supportive care.^3,4^

In this context, demonstration of the efficiency of sorafenib, a multikinase inhibitor, was an important milestone in the treatment of patients with advanced HCC. The SHARP pivotal trial^5^ and its validation in Asia^6^ have shown that sorafenib delays tumour progression and improves overall survival (OS), but its ability to induce tumour shrinkage is very modest. Sorafenib is widely approved as the standard first-line treatment for advanced HCC patients. Numerous phase III trials of various molecular-targeted agents vs. sorafenib have been conducted, but none has shown, so far, a superior survival benefit to sorafenib.^7^ An alternative treatment to sorafenib monotherapy was needed then. Since then, a recent phase III study has shown the non-inferiority of lenvatinib compared with sorafenib monotherapy, which can be a new option for first-line treatment.^8^ According to the clinical recommendations,^9^ cytotoxic chemotherapy is not considered as a therapeutic option; for patients intolerant to sorafenib or for whom sorafenib and regorafenib have failed, inclusion in a clinical trial or supportive cares is indicated. New second-line options are awaited, including cabozantinib with a recent positive phase III trial,^10^ ramucirumab^11^ and nivolumab, with interesting phase I–II results^12^ and an ongoing phase III study (CheckMate-459 NCT02576509).

Oxaliplatin-based regimens in the treatment of advanced HCC have been evaluated in phase I and II trials.^13–16^ In France, the gemcitabine plus oxaliplatin (GEMOX) regimen appeared to be one of the most promising, with low renal and hepatic toxicity in cirrhotic patients and encouraging efficacy results in phase II trials.^17,18^ In a recent large retrospective study, the GEMOX combination achieved an encouraging overall response rate of 22% and an OS of 11 months.^19^ Based on these encouraging results in the literature, we conducted a randomised phase II study to evaluate the efficacy and safety of the sorafenib plus GEMOX combination as first-line treatment for patients with advanced unresectable or metastatic HCC.

## Methods

### Study design and patients

We conducted an open-label multicentre phase II randomised study. Patients from ten centres were included. Patients with histologically documented advanced or metastatic HCC (i.e., non-resectable, non-transplantable and non-accessible to percutaneous ablation) were eligible. Patients where either non-eligible for chemoembolization or were included after failure of chemo-embolization. They were included in the study if they had at least one measurable target lesion not previously treated with arterial chemoembolization, radiofrequency, alcohol or cryoablation. Other main eligibility criteria were World Health Organization (WHO) performance status ≤ 1, age ≥ 18 years, life expectancy ≥ 12 months, Cancer of Liver Italian Progression (CLIP) score ≤ 3,^20^ absence of known brain metastasis, encephalopathy and ascites, negative pregnancy test and effective contraception. The following biology criteria were also requested: absolute neutrophil count ≥ 1 500 cells/µL; haemoglobin ≥ 9 g/dl, platelet count ≥ 90 000 cells/µL, serum creatinine < 1.5 times the upper limit of normal (ULN), creatinine clearance according to the Cockroft and Gault formula ≥ 60 mL/min; serum albumin ≥ 28 g/L; international normalised ratio ≤ 2.3; prothrombin time ≥ 40%; serum bilirubin ≤ 1.5 × ULN and serum transaminases ≤ 5 × ULN. Patients were excluded if they had previously been treated with systemic chemotherapy or antiangiogenic therapy for their HCC, stage B or C cirrhosis according to the Child Pugh classification, or if they were concomitantly treated with any other anticancer treatment (including tamoxifen, interferon and somatostatin analogues) or any strong CYP3A4 inducer; if they had history of epilepsy or organ graft associated with immunosuppressive agents, or of other cancer (aside from basocellular skin tumours and appropriately treated cervical cancer). Other ineligibility criteria included active and severe bacterial or fungal infection, known HIV infection, exclusive bone metastasis, grade ≥ 2 peripheral neuropathy (according to the National Cancer Institute Common Terminology Criteria for Adverse Events–NCI CTCAE version 3.0), allergy to experimental treatment, serious cardiac disorders (e.g., arrhythmia needing a treatment, recent history of myocardial infarct, unstable hypertension, etc.), intestinal malabsorption, obstruction syndrome, dysphagia, ongoing pregnancy or breastfeeding. All patients signed an informed consent before entering the study. The study protocol was approved by an ethical review board and conducted in accordance with the good clinical practice and Declaration of Helsinki guidelines (Clinical trial ID: NCT00941967).

**Fig. 1:**
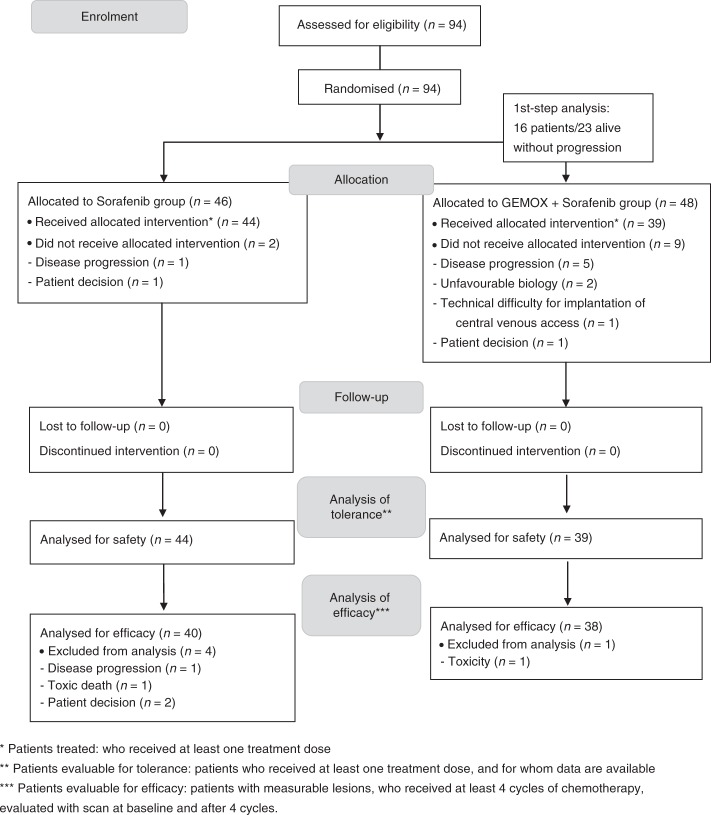
CONSORT flow diagram of the study

Patients were randomly and equally allocated (ratio 1:1) to one of the two treatment arms (i.e., sorafenib alone or GEMOX plus sorafenib, Fig. 1) using a centralised, randomised block design, with random block size option. Assignment was stratified according to the CLIP score (0–1 vs. 2–3).

### Objectives

The primary objective of the study was to assess the progression-free survival (PFS) rate at 4 months in the experimental arm (sorafenib plus GEMOX combination). Secondary objectives included evaluation of the safety of the experimental regimen, objective response to treatment, disease control rate, median PFS and median OS.

### Treatments and outcome assessments

Patients in the two arms, sorafenib alone and sorafenib plus GEMOX, were to receive 400 mg of oral sorafenib twice daily, starting on day 1. This study drug was provided by Bayer Health Care. Gemcitabine (1000 mg/m^2^) was delivered intravenously over 100 min on day 1 and 100 mg/m^2^ oxaliplatin were administered as a 2 h infusion on day 2, every 2 weeks (day 1 = day 14). A treatment cycle was defined as 28 treatment days comprising two GEMOX courses. The treatment was administered for 6 cycles (12 GEMOX courses) or until disease progression, or occurrence of a limiting toxicity.

Tumour evolution was monitored by computed-tomography scan (± hepatic magnetic resonance imaging) according to the RECIST 1.0 (Response Evaluation Criteria in Solid Tumours) every 8 weeks until progression. A second radiologic review was performed retrospectively using the mRECIST (modified RECIST) criteria. Toxicity events were graded according to the NCI CTCAE (version 3.0).

### Pharmacokinetics

Optionally, blood samples on lithium heparinate were collected every 2 weeks before administration of sorafenib. Plasma was frozen at −80 °C until centralised sorafenib high-performance liquid chromatography analysis.

### Statistical considerations

The primary endpoint was the 4-month PFS rate in the sorafenib plus GEMOX arm. PFS was defined as the time between the beginning of treatment to the first progression or death in case of progression (failure). A total of 78 patients were required, 39 in each arm, according to a Simon two-stage minimax design with α = 10% and β = 10% (p0 = 0.50 and p1 = 0.70). Twenty-three patients were required for the first stage. The trial was to continue to the second stage after an analysis at the end of the first stage, if 12 or more patients of the first stage were alive without progression at 4 months. The second stage required 39 patients and the experimental regimen was to be considered promising if 24 or more patients were alive without progression at 4 months. Secondary endpoints were tolerance, response to the treatment and OS. PFS and OS were estimated using the Kaplan–Meier method. Patients were evaluable for tolerance and efficacy if they had received at least one course and four cycles of treatment, respectively.

Categorical variables were reported by means of contingency tables and continuous variables using medians and ranges. To investigate their associations with the clinical, pathologic, and biologic parameters, univariate statistical analyses were performed using the Pearson’s *χ*^2^-test or Fisher’s exact test when applicable for categorical variables, and using the Kruskal–Wallis test or Student’s *T*-test for continuous variables. PFS rates (event: the first observation of tumour progression or death) and OS rates (event: death from any cause) were estimated using the Kaplan–Meier method and presented with their 95% confidence intervals (95% CIs). Survival curves were drawn and the log-rank test was performed to assess the difference between the two groups. Patients alive without event were censored at last news date. The median follow-up was estimated using the reverse Kaplan–Meier method and presented with its 95% CI. All statistical analyses were performed using the STATA11 software (Texas, USA).

## Results

### Patients

A total of 94 patients were assessed for eligibility and were included in the study between December 2008 and October 2011 (Fig. 1). Patients were allocated to the control arm (sorafenib alone, *n* = 48) or the experimental arm (sorafenib plus GEMOX, *n* = 46). The stratification was adequately performed as CLIP scores were well-balanced between the two arms, as well as other baseline characteristics (Table 1).

**Table 1:** Baseline characteristics in randomly assigned patients

	Sorafenib (*n* = 44)	GEMOX + sorafenib (*n* = 39)
Age (years), median [range]	62 [39–78]	65 [47–79]
Sex, *n* (%)		
Male	38 (86%)	36 (92%)
Female	6 (14%)	3 (8%)
WHO performance status, *n* (%)		
0	31 (70%)	28 (72%)
1	13 (30%)	11 (28%)
CLIP score, *n* (%)		
0–1	20 (46%)	20 (51%)
2–3	24 (54%)	19 (49%)
BLCC score, *n* (%)		
B	8 (18%)	4 (10%)
C	36 (82%)	35 (90%)
Underlying cirrhosis, *n* (%)	26 (62%)	23 (62%)
Disease aetiology, *n* (%)		
Hepatitis B	1 (2%)	2 (5%)
Hepatitis C	6 (14%)	7 (18%)
Alcohol	15 (34%)	10 (26%)
NASH	13 (30%)	15 (39%)
Others	9 (20%)	5 (13%)
Portal vein thrombosis, *n* (%)	11 (25%)	11 (28%)
Previous anticancer treatment, *n* (%)	24 (55%)	22 (56%)
Surgery	15 (34%)	11 (28%)
TACE	13 (30%)	11 (28%)
Radiofrequency ablation	5 (11%)	8 (21%)
Disease status, *n* (%)		
Liver limited disease	17 (39%)	9 (23%)
Extra-hepatic disease	27 (61%)	30 (77%)
Serum α-fetoprotein level (µg/L), median [range]	25.7 [0.9-140 300]	70.4 [1.5-59455]

*BCLC* Barcelona Clinic Liver Cancer, *CLIP* Cancer of Liver Italian Progression, *NASH* non-alcoholic steatohepatitis, *TACE* transarterial chemoembolization, *WHO* World Health Organization

### Treatments

The median duration of the sorafenib treatment was 4 months (range: 1–51) in both arms (Supplementary Table 1). The calculated relative dose intensity (RDI) revealed that 19 patients (43%) and 23 patients (59%) received 80% or more of the sorafenib recommended dose in the sorafenib alone and sorafenib plus GEMOX arms, respectively, which was not significantly different between the two groups. The sorafenib RDI was 75% in the sorafenib arm and 84% in the sorafenib plus GEMOX arm (Supplementary Table 1). Non-compliance (63% of non-compliant patients) was mostly due to haematological and neurologic toxicity. The median number of GEMOX courses administered to patients in the experimental arm was 7 (range: 1–16). The relative dose intensities for the combination treatments were respectively of 78% for gemcitabine and 68% for oxaliplatin.

Fifty-five per cent of patients (*n* = 24) of the sorafenib arm received second-line treatment, as compared with only 15% (*n* = 6) of the sorafenib plus GEMOX arm (who have been treated with oral fluoropyrimidine or doxorubicin in a non-trial setting, data not shown).

### Toxicity

Patients in the sorafenib and sorafenib plus GEMOX arms globally experienced comparable severe toxicities (Table 2). As expected, more haematological and sensitive neuropathy events occurred in the sorafenib plus GEMOX arm, whereas more patients in the sorafenib arm presented a hand–foot syndrome. Main significant severe toxicities were (control vs. experimental arm): neutropenia (grade 3–4: 0% vs. 23%), fatigue (grade 3: 7% vs. 21%), thrombocytopenia (grade 3: 0% vs. 33%), diarrhoea (grade 3: 9% vs. 18%), peripheral neuropathy (grade 2–3: 0% vs. 5%), and hand–foot syndrome (grade 3: 18% vs. 5%). One patient in the sorafenib alone arm died within the month after the end of treatment, following a severe adverse event (*Pneumocystosis carinii* pneumonia).

**Table 2:** Main toxicities in the sorafenib and sorafenib plus GEMOX arms

	Sorafenib (*n* = 44)		GEMOX + sorafenib (*n* = 39)		*p*-Value^a^
	Grades 1–2	Grades 3–4	Grades 1–2	Grades 3–4	
Overall toxicity, *n* (%)	11 (25%)	32 (73%)	8 (21%)	31 (79%)	0.472
Asthenia, *n* (%)	28 (64%)	3 (7%)	26 (67%)	8 (21%)	0.066
Nausea/vomiting, *n* (%)	23 (52%)	18 (41%)	22 (56%)	13 (33%)	0.476
Diarrhoea, *n* (%)	29 (66%)	4 (9%)	25 (64%)	7 (18%)	0.235
Neutropenia, *n* (%)	7 (16%)	0	17 (44%)	9 (23%)	**<** **0.001**
Thrombocytopenia, *n* (%)	22 (50%)	0	21 (54%)	13 (33%)	**<** **0.001**
Cutaneous, *n* (%)	31 (70%)	10 (23%)^b^	24 (62%)	5 (13%)^b^	0.242
Hand-foot syndrome, *n* (%)	29 (66%)	8 (18%)^b^	16 (41%)	2 (5%)^b^	0.068
Sensitive neuropathy, *n* (%)	0	0	20 (51%)	2 (5%)^b^	**0.029**

^a^Difference of grade 3–4 toxicity occurrence between the two arms (*χ*^2^-test)

^b^Only grade 3 for cutaneous toxicities (hand–foot syndrome and sensitive neuropathy)

### Efficacy: tumour response

Among the 83 patients who received treatments, 5 patients were not evaluable for efficacy, 4 in the sorafenib arm and 1 in the sorafenib plus GEMOX arm (Fig. 1). At the first-stage analysis, 16/23 patients evaluable in the experimental arm were not progressive at 4 months. The objective response rate, according to the RECIST criteria, was of 9% (90% CI: 3–20) in the sorafenib arm and 15% (90% CI: 6–28) in the sorafenib plus GEMOX arm (Supplementary Figure 1). The disease control rate was 70% (90% CI: 57–81) and 77% (90% CI: 63–87) in the sorafenib alone and sorafenib plus GEMOX arms, respectively. No complete response was observed. One patient in the sorafenib arm was resected after 12 months and another patient in the same treatment arm was still alive and non-progressive after 50 months. Using the mRECIST criteria, as expected, more partial responses were reported. The objective response rate was 20.5% (90% CI: 9–35) and 28.2% (90% CI: 15–45) in the sorafenib alone and sorafenib plus GEMOX arms, respectively.

### Efficacy: survival

**Fig. 2:**
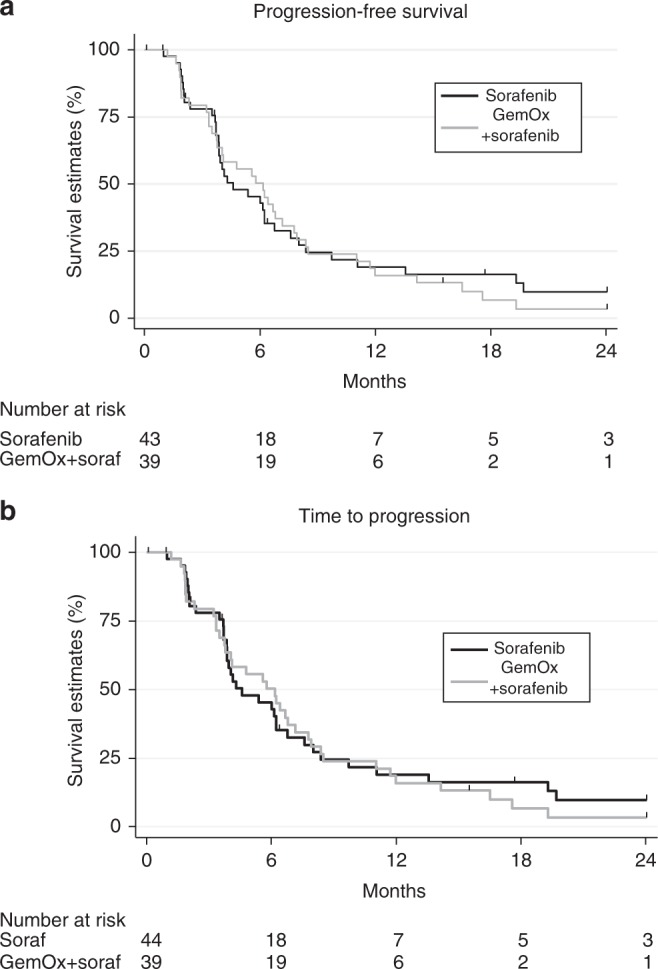
Progression-free survival (**a**) and time-to-progression (**b**) according to the treatment arm

The median follow-up was 36.8 months for patients in the sorafenib arm and 44.4 months for patients in the sorafenib plus GEMOX arm. At the time of analysis, 67 patients (81%) had died, 32 (73%) in the sorafenib arm and 35 (90%) in the sorafenib plus GEMOX arm. The main cause of death was disease progression (56 patients, 84%). The PFS rate at 4 months was 64% in the sorafenib plus GEMOX arm (Table 3), compared with 61% in the sorafenib arm. According to the design of the trial, the sorafenib plus GEMOX combination met its primary endpoint. The median PFS was 4.6 (90% CI: 3.9–6.2) months in the sorafenib arm and 6.2 (90% CI: 3.8–6.8) months in the combination arm (Fig. 2). The median time to progression was 4.6 months (95% CI: 3.8–6.2) and 6.2 (95% CI: 3.7–7.2) in the sorafenib alone vs. sorafenib plus GEMOX arms, respectively (Fig. 2). The median OS was 14.8 (90% CI: 12.2–22.2) months and 13.5 (90% CI: 7.5–16.2) months in the sorafenib and sorafenib plus GEMOX arms, respectively (Table 3).

**Table 3:** Best tumour response by RECIST 1.0 and survival

	Sorafenib (*n* = 44)	GEMOX + sorafenib (*n* = 39)
Progression-free survival		
At 4 months, *n* (%)	27 (61%)	25 (64%)
Median (months), [90% CI]	4.6 [3.9–6.2]	6.2 [3.8–6.8]
Best response to treatment, *n* (%)		
Partial response	4 (9%)	6 (15%)
Stable disease	27 (61%)	24 (62%)
Progressive disease	9 (21%)	8 (21%)
Non-assessable	4 (9%)	1 (3%)
Disease control rate, % [90% CI]	71 [57–81]	77 [63–87]
Overall survival, median (months) [90% CI]	14.8 [12.2–22.2]	13.5 [7.5–16.2]

*CI* confidence interval

### Exploratory analysis

In an exploratory analysis, OS in the sorafenib plus GEMOX arm was analysed according to the presence or absence of cirrhosis, the tumour location (liver-only, i.e., intra-hepatic exclusively vs. extra-hepatic disease, i.e., intra and extra-hepatic, or extra-hepatic disease only) and according to serum α-fetoprotein (Table 4). None of these factors could identify a significant difference for OS in these subgroups of patients. However, patients with intra-hepatic lesions only, cirrhosis or α-fetoprotein ≥ 200 (µg/L) had a trend to a shorter OS. Indeed, median OS was 6.9 months (95% CI: 3.0–13.7) for patients with liver-only disease (*n* = 9) compared with 14.3 (95% CI: 7.8–19.1) for patients with extra-hepatic disease (*n* = 30); it was 8.4 months (95% CI: 6.9–15.5) vs. 17.6 months (95% CI: 7.2–23.3) in patients with cirrhosis (*n* = 23) vs. patients without cirrhosis (*n* = 16), and 7.8 months (95% CI: 6.3–13.5) compared with 17.6 months (95% CI: 8.4–22.3) in patients with serum α-fetoprotein ≥ 200 µg/L (*n* = 23) or < 200 µg/L (*n* = 15), respectively.

**Table 4:** Survival analysis in the GEMOX plus sorafenib arm according to cirrhosis, extra-hepatic spread and αfetoprotein (αFP) level

GEMOX + sorafenib (*n* = 39)	PFS months (95% CI)	*p*-Value^1^	OS months (95% CI)	*p*-Value^a^
All evaluable patients	6.2 (3.8–6.8)		13.5 (7.5–19.1)	
Cirrhosis		0.866		0.736
With cirrhosis	6.2 (1.9–7.2)		8.4 (6.9–15.5)	
Without cirrhosis	6.2 (3.8–8.4)		17.6 (7.2–23.3)	
Disease status		0.486		0.275
Extra-hepatic disease	6.2 (3.8–7.9)		14.3 (7.8–19.1)	
Liver-only disease	4.1 (3.2–6.8)		6.9 (3.0–13.7)	
α-Fetoprotein level (µg/L)		0.347		0.065
Serum α-fetoprotein < 200 µg/L	6.2 (4.0–8.4)		17.6 (8.4–22.3)	
Serum α-fetoprotein ≥ 200 µg/L	6.2 (3.4–7.2)		7.8 (6.3–13.5)	

*OS* overall survival, *PFS* progression-free survival

^a^Correlation between cirrhosis, extra-hepatic spread, αFP and survival using the log-rank test

For the subgroup of patients who underwent 6 months of treatment (in our study, 34 patients, 17 in each arm), the median PFS and OS in the sorafenib plus GEMOX arm were 11.0 (90% CI: 6.7–14.2) and 19.1 months (90% CI: 15.5–25.7), respectively. For patients treated in the sorafenib plus GEMOX arm, the median OS was 12.8 months.

### Pharmacokinetics

No difference was observed between the sorafenib and the sorafenib plus GEMOX arms regarding sorafenib pharmacokinetics data (Supplementary Table 2).

## Discussion

Our results show the feasibility of the addition of GEMOX to sorafenib in advanced HCC patients. The PFS rate at 4 months was 64% in the sorafenib plus GEMOX arm. According to the trial design, the sorafenib-GEMOX combination can thus be considered effective. The median PFS was 6.2 months (range: 3.8–6.8) in the sorafenib plus GEMOX arm and the median OS was 13.5 months (range: 7.5–19.1). These survival results obtained in a poor prognostic population (extra-hepatic metastasis, 77%; portal vein thrombosis, 28%; and CLIP score ≥ 2, 49%) are comparable as compared with those of the literature.^5,6^

In a similar setting, a recent phase II trial of sorafenib plus doxorubicine combination seemed promising,^21^ but results of the combination in a larger cohort in a phase III trial just showed a lack of expected benefit.^22^ Results of other large first-line studies have been published these last years, often negative,^23–25^ which confirmed sorafenib alone as the keystone treatment in first-line and the lack of predictive factor of response.

We have studied the impact of the treatment in subgroups of diverse prognosis. As seen elsewhere,^26^ underlying cirrhosis and a high α-fetoprotein rate were correlated with poorer survival. Surprisingly, a trend for higher PFS (6.2 vs. 4.1 months) and OS (14.3 vs. 6.9 months) was found in patients with extra-hepatic disease in the sorafenib plus GEMOX arm. This was not found in patients treated with sorafenib alone. It could constitute a potential hypothesis for further development of this treatment combination during which chemotherapy is used as ‘starter’ and sorafenib is then administrated alone in responder patients.

In our study, the objective response and disease control rates were of 15% and 77%, respectively, in the sorafenib plus GEMOX arm. Although cross-study comparisons should be made with caution, an objective response rate of 15% compares favourably with that observed with vascular endothelial growth factor receptor inhibitors alone (2–6%),^5,6^ but also with that of the sorafenib plus doxorubicin combination (4%).^19^ This may be of clinical value, notably for downstaging locally advanced tumours, which may allow subsequent curative-intent therapies.

As no predictive factor yet predicts response to sorafenib treatment, any patient stratification that could have helped select patients who may benefit from the addition of GEMOX cytotoxic regimen was not possible. However, a single arm prospective study has asked the question of the potential benefit of the sorafenib and GEMOX combination in 49 patients.^27^ After 6 cycles of GEMOX plus sorafenib, 25 patients have continued sorafenib alone. Overall, in this selected population (responder patients with no limiting toxicity to sorafenib), the time to progression was 10.3 months and the median OS was 15.7 months.^27^ In our study, survival figures are comparable for patients who have undergone 6 months treatment

Our results in terms of overall response rate suggest a possible additional ‘starter’ effect of this cytotoxic regimen when associated with sorafenib. Nevertheless, they argue against giving up such a cytotoxic regimen within the therapeutic landscape of HCC in selected cases of locally advanced HCC. In addition, the known immunogenic death induced by oxaliplatin^28^ may synergise the anti-tumour activity of innovative immune-oncology agents, such as tremelimumab and nivolumab, which appear promising in HCC.^12,29^ Such treatment combinations and sequencing could be interesting to explore as to initiate a tumour and inflammatory response before immune therapy start.^30,31^

In the pharmacokinetics analyses, no significant difference was found between the two arms, especially in terms of residual sorafenib concentration, which could have explained our data. Contrasting with the decreased sorafenib dose intensity observed in previous studies of doxorubicine plus sorafenib combination,^20^ in our study, patients received a similar number of treatment cycles and a comparable sorafenib dose intensity in the two arms. Our data regarding patients treated with sorafenib are comparable to those of the literature.^5,6^ Regarding the GEMOX regimen, patients received a median of seven cycles, which is also comparable to other previously published studies in HCC patients receiving first-line treatment.^13,19^ Adverse effects of GEMOX plus sorafenib were essentially additive and similar to what would be expected for each of these drugs used as a single agent. Dose reductions or treatment discontinuations of GEMOX were mainly due to haematologic toxicity, which was expected in these patients who are often cirrhotic (33% of grade 3–4 thrombopenia and 23% of grade 3–4 neutropenia). We did not report more cutaneous nor digestive toxicities, or unexpected adverse events in the sorafenib plus GEMOX arm compared with the sorafenib alone arm. The grade 2 neurotoxicity rate was quite low. In addition, no toxic death was reported in the sorafenib plus GEMOX arm. Taken together, these results suggest the absence of clear pharmacologic drug interactions between sorafenib and GEMOX.

One limitation of our study for interpreting OS was the fact that more patients in the sorafenib alone arm have received, in the context of innovative therapeutic trials, a second-line treatment.^25–31^ This could account for the lack of difference in OS between the two arms and the unexpectedly high OS in the sorafenib arm (median OS of 13 months, longer than that of most control arms of previously published studies). The potential impact of the second-line treatments in terms of OS favouring the sorafenib arm was especially true in patients who have received at least six sorafenib cycles (data not shown). In those patients, median OS in the sorafenib arm was 25.3 months (95% CI: 9.0–30.5), whereas the treatment was discontinued for more than 6 months and no second-line treatment validated yet. For patients in the sorafenib plus GEMOX, the median OS was 19.1 months (95% CI: 8.5–32.5), which underlines the lack of therapeutic resources and poor prognostic possibly related to the impossibility to include these patients in a second-line trial.

## Conclusion

Our study shows the feasibility of the sorafenib and GEMOX combination. The toxicity, moderate benefit on PFS and the lack of predictive factors of response to the sorafenib and GEMOX combination make a subsequent phase III study not justified in unselected HCC patients. Although our study is positive for its primary endpoint, we cannot recommend this combination of chemotherapy with sorafenib as standard of treatment, all the more now that new options are available in the landscape of HCC treatments, in first (lenvatinib) or second line (cabozantinib and ramucirumab). Results of the ongoing or future clinical trials, especially those assessing immunotherapies, are eagerly awaited.

## Supplementary information

Supplemental figures and tables
