# Supplementary material for: Sorafenib alone vs. sorafenib plus GEMOX as 1st-line treatment for advanced HCC: the phase II randomised PRODIGE 10 trial
Source: Br J Cancer. 2019 Apr 4;120(9):896–902. doi: 10.1038/s41416-019-0443-4 (PMC6734663; doi:10.1038/s41416-019-0443-4)
Supplement: Supplementary file 1 — Supplemental figures and tables [file 41416_2019_443_MOESM1_ESM.docx]

**Supplementary Table 1:** Treatments and dose intensity

|  | **Sorafenib (n=44)** | **GEMOX + sorafenib (n=39)** |
| --- | --- | --- |
| Number of sorafenib cycles, median [range] | 4 [1-50] | 4 [1-27] |
| Treatment duration (months), median [range] | 3.9 [0.9-51.2] | 4.1 [0.9-27.4] |
| Sorafenib dose intensity (mg/j) | 600 | 669 |
| Sorafenib RDI (%), median [range] | 75 [2-106] | 84 [11-125] |
| < 80%, n (%) | 25 (57%) | 16 (41%) |
| ≥ 80%, n (%) | 19 (43%) | 23 (59%) |
| Number of GEMOX courses, median [range] | - | 7 [1-16] |
| Treatment duration (weeks), median [range] | - | 15 [2-34] |
| Gemcitabine RDI (%), median [range] | - | 78 [40-105] |
| Oxaliplatin RDI (%), median [range] | - | 68 [36-106] |
| DI: Dose intensity; RDI: Relative Dose Intensity | | |

**Supplementary Table 2:** Baseline characteristics in randomly assigned patients

| **Sorafenib  (n=44)** | | | **GEMOX + sorafenib**  **(n=39)** | | |
| --- | --- | --- | --- | --- | --- |
| Patient  number | Residual Soraf concentration (mg/L) | Daily sorafenib dose (mg/day) | Residual Soraf concentration mg/L | Daily sorafenib dose (mg/day) | Patient  number |
| 1 | 8.91 | 800 | 7.31 | 800 | 2 |
| 5 | 5.3 | 800 | 7.19 | 800 | 3 |
| 6 | Not done | | 7.63 | 400 | 4 |
| 7 | Not done | | 5.91 | 800 | 8 |
| 10 | Not done | | 7.92 | 800 | 11 |
| 13 | 2.18 | 800 | 2.73 | 800 | 12 |
| 15 | 11.6 | 800 | 3.6 | 800 | 14 |
| 18 | 10.53 | 800 | 2.79 | 800 | 22 |
| 19 | 7.78 | 600 | 4.19 | 400 | 24 |
| 20 | 10.17 | 800 | 1.91 | 400 | 25 |
| 23 | Not done | | 3.47 | 800 | 29 |
| 27 | 6.17 | 800 | 7.65 | 800 | 31 |
| 28 | 8.65 | 800 | Not done | | 32 |
| 30 | 12.42 | 800 | Not done | | 36 |
| 34 | 3.65 | 800 | Not done | | 38 |
| 35 | Not done | | 1.46 | 800 | 42 |
| 37 | 14.16 | 600 | 2.69 | 800 | 45 |
| 39 | 3.57 | 800 | 4.03 | 800 | 51 |
| 40 | Not done | | 8.11 | 600 | 53 |
| 44 | 2.17 | 800 | Not done | | 55 |
| 46 | 7.85 | 800 | Not done | | 56 |
| 48 | 7.24 | 800 | 2.57 | 800 | 58 |
| 49 | 7.54 | 800 | Not done | | 61 |
| 50 | Not done | | Not done | | 64 |
| 52 | 2.7 | 400 | Not done | | 66 |
| 54 | Not done | | Not done | | 68 |
| 57 | Not done | | Not done | | 69 |
| 59 | Not done | | Not done | | 73 |
| 60 | 12.94 | 800 | Not done | | 74 |
| 63 | Not done | | 3.22 | 800 | 79 |
| 65 | Not done | | 6.99 | 800 | 81 |
| 67 | 5.6 | 800 | Not done | | 82 |
| 70 | 5.97 | 600 | 7.54 | 800 | 83 |
| 71 | Not done | | 6.41 | 800 | 86 |
| 72 | 3.49 | 400 | Not done | | 87 |
| 75 | Not done | | Not done | | 88 |
| 76 | 9.73 | 600 | 11.27 | 800 | 89 |
| 77 | 4.05 | 400 | 1.74 | 800 | 90 |
| 78 | 6.96 | 800 | 3.44 | 800 | 91 |
| 80 | Not done | |  |  |  |
| 84 | Not done | |  |  |  |
| 92 | Not done | |  |  |  |
| 93 | Not done | |  |  |  |
| 94 | 7.91 | 800 |  |  |  |

**Supplementary Figure 1:** Waterfall plot – Best tumour response

Patients

-100

-80

-60

-40

-20

0

20

40

60

Best tumour response (%)
